# Common differential diagnosis of low back pain in contemporary medical practice: a narrative review

**DOI:** 10.3389/fmed.2024.1366514

**Published:** 2024-02-06

**Authors:** Dilyan Ferdinandov, Dimo Yankov, Martin Trandzhiev

**Affiliations:** ^1^Department of Neurosurgery, Medical University of Sofia, Sofia, Bulgaria; ^2^Clinic of Neurosurgery, St. Ivan Rilski University Hospital, Sofia, Bulgaria; ^3^Faculty of Medicine, Medical University of Sofia, Sofia, Bulgaria

**Keywords:** low back pain, differential diagnosis, mechanical pain, non-mechanical pain, referred pain

## Abstract

With a wide range of etiologies, low back pain (LBP) presents a true clinical challenge, finding its origins both in intrinsic spinal and systemic conditions, as well as referred ones. This review categorizes the LBP into these three groups and aims to offer a comprehensive look at the tools required to diagnose and differentiate them. The intrinsic etiologies are based on conditions that affect the musculoskeletal components of the lumbar spine, such as intervertebral disc disease, stenosis, muscular imbalance, and facet joint degeneration. The systemic causes usually extend beyond local structures. Such are the cases of neoplasia, infections, and chronic inflammation. The diagnosis is rendered even more complex by adding the referred pain, which only manifests in the lower back yet arises in more distant locations. By synthesizing the literature that encompasses the problem, this review aims to augment the understanding of the differential diagnoses of LBP by showcasing the subject’s nuances. This categorization provides a structured approach to a patient-centered diagnosis, which could facilitate the medical practitioners’ efforts to navigate this pathology more effectively.

## Introduction

Low back pain (LBP) is one of the most frequently observed symptoms in the general population, with the most disability-adjusted life years, as well as an impact on the economy and social state of the affected patients (1). According to a systemic analysis of the global burden, at the beginning of the decade, about 619 million people were affected, with a projection of 843 million prevalent cases in the middle of the century (2). The intensity of the pain correlates with decreased overall productivity of the individual and a loss of the ability to function normally. The condition is defined by the identification or lack thereof of a nociceptive cause and is thus divided into specific and non-specific LBP. In the cases of directly related causes of LBP, an actual pathoanatomical substrate can be identified on imaging and is accompanied by a medical history, such as a presence of comorbidities (e.g., in the case of a metastasis) or a preceded trauma in the spinal fracture cases. In non-specific LBP, the pain is not easily attributed to either category, and a certain conclusion for the actual reason for the pain is impeded (3).

The condition is further classified according to its source, which, when correctly identified, is most commonly the result of a disturbance of the structures of the particular spine. These might be the discs, the vertebra, or the associated ligamentous or joint tissues, in which case it is accepted as a mechanical or an intrinsic spinal condition (4). Additionally, pain that is strongly associated with the spine, however, is not directly caused by damage to either of the structures but rather by a process that secondarily involves them, such as an infection or metastatic neoplasia, is referred to as a non-mechanical or systemic condition (5, 6). Furthermore, the LBP experienced in certain patients could be from a completely different origin and not connected to the spinal cord or its structures. Such is the case in the so-called referred pain, which typically arises from a visceral organ or has a pelvic origin (7). In some instances, the diagnosis is still complicated, accounted for by the wide range of factors that could contribute to the symptomatology (1, 3).

We aim to present the entirety of the conditions associated with low-back pain and the methods used to diagnose them and differentiate them from the rest of the diagnoses. Some of them have well-known causes with a large number of reported studies and case series. Others are less frequently noted, mainly through singular case reports or limited studies. Having a more comprehensive look at all of the factors involved in LBP could help reduce the number of misdiagnoses and subsequently lower the socio-economic burden of the condition.

## Materials and methods

This study conducted a comprehensive narrative review on the PubMed database, which offers extensive biomedical literature content. The strategy included a search for “low back pain causes,” which gave us a more general look at the conditions associated with our aim. A total of 20 381 results were found. Later, a combination of the condition’s name followed by the term “low back pain” was conducted for each identified condition to acquire a more comprehensive look into the cases and studies published regarding the individual conditions.

The inclusion criteria focused on human studies written in English, with neither clinical nor experimental studies being excluded, as long as they were relevant to understanding the etiology of low back pain. We were not interested on case reports and reviews. The studies were not limited by year of publication, with the idea of also examining more rare conditions that are not frequently published about. Nevertheless, we focused on the papers in the last 5°years, which were reduced to 4,977 for the period. At the end, a list was compiled of 257 publications.

The data was extracted to identify the key themes and contributors to the presence and exacerbation of low back pain. The anatomical substrate of the pain was also identified and commented on where possible. We designed flowcharts that allow for a coherent presentation of the diverse range of causes of low back pain.

## Mechanical or intrinsic spinal conditions

**FIGURE 1:**
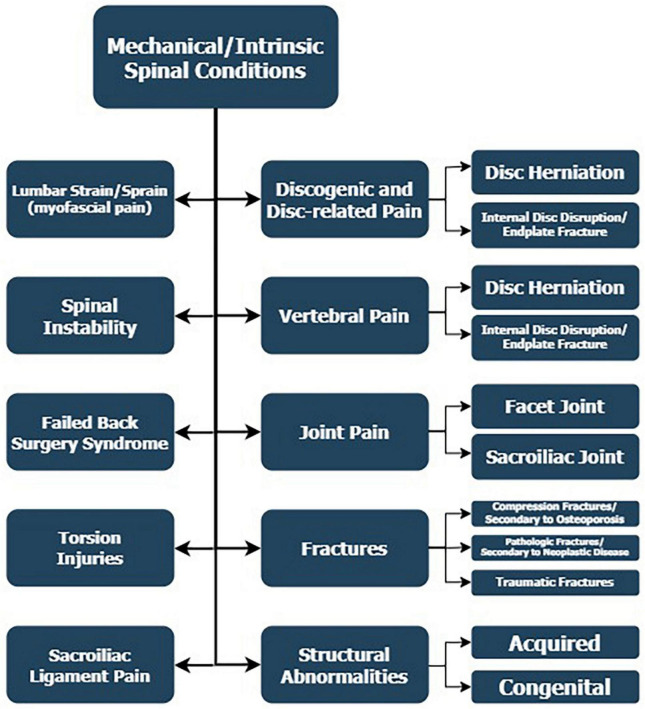
Mechanical or intrinsic spinal conditions from the anatomical components of the spine and pathological developments that could account for LBP.

The mechanical conditions of the spine account for the predominant number of cases of low back pain (Figure 1). There are many anatomical components and pathological developments that could potentially account for LBP with mechanical genesis.

### Discogenic and disc-related pain

The discogenic pain is usually attributed to intrinsic disc degeneration or an endplate fracture. The degeneration of the intervertebral disc is an event of a non-complete annular tear by the disc’s nucleus, and it usually produces pain along the sinuvertebral nerves, innervating the disc (8). The pathology is experienced as a dull ache in symptomatic cases and is diagnosed through an MRI or a provocating discography. The latter of the methods relies on the replication of the pain with an increase in the intradiscal pressure. Additionally, an endplate fracture could cause LBP, seeing as patients with histologically verified micro-fractures of the endplate present with significantly higher pain and disability scores than normal (9).

On the other hand, disc-related pain results from conditions such as nucleus pulposus herniation, where the disc itself and its innervation are not the primary cause of the complaints. They provoke a radiating pain through the compression of a nerve root. The distribution of the pain is in three main patterns – low back, buttock, and leg radiating pain. The gold standard for diagnosing the condition is the MRI, showing T2-weighted signal changes confirming disc herniation (10).

### Lumbar strain/sprain

The pain in this subgroup of patients originates from the muscles or fascia of the lower back. The term strain describes an excessively stretched or torn muscle, while a sprain is the process of ligament tearing (11). The conditions are usually associated with an acute injury or gradual wearing out of the related structure. The diagnosis is traditionally made with an MRI to examine soft tissues better. The CT is an important tool especially for initial work-up in traumatic cases. Yet, an X-ray could be used as a cheaper tool for the differential diagnosis of a vertebral fracture or a local infection but with lower sensitivity. A direct relationship was indicated between the elastic coefficient of the thoracolumbar fascia and the degree of LBP experienced by a group of people, with higher pain levels associated with lower elastic coefficients (12).

### Vertebral pain, traumatic and compression fractures

The vertebral pain arises from the vertebral body or the posterior elements, such as pedicles and laminae. In the context of a mechanical condition, the cause is usually a traumatic or a compression fracture (13, 14). As for the vertebral body, a compression fracture is the most common cause, typically resulting from a fall. Osteoporotic patients are especially endangered since the bone density is low and the axial force required to induce a fracture is minimal. The diagnosis is based primarily on the physical examination, involving a history of a fall or trauma, combined with risk factors, such as glucocorticoid use and osteoporosis, as well as LBP and loss of height (13). Even though the diagnosis is confirmed through a simple radiograph, a large number of cases remain undiagnosed. Nowadays, the employment of deep-learning algorithms in medicine has the potential to facilitate the process of detection and assessment of compression fractures using X-rays. Algorithms demonstrate results superior to those of trainee radiologists, on par with expert radiologists. Thus, the novel technology could potentially facilitate the diagnosis in primary medical centers (14).

### Facet and sacroiliac joint pain

As for facet joint pain, the primary substrate is the medial branch of the posterior rami of the respective spinal nerve and the one just above the engaged zygapophyseal joint. The pain is most usually unilateral and limited in irradiation from the joint of origin until the buttocks, more rarely moving down the thigh and mimicking radiculopathy. The cause is often an underlying degenerative process (15). Osteoarthritis, as a degenerative disease of the joints, has been linked with a higher incidence of LBP among patients operated on for disc herniation, as presented by Chen et al. (15). The authors noted that non-bacterial joint inflammation should be more meticulously examined pre-op since the condition could hinder the actual resolution of the lumbar pain (15).

Additionally, pain arising from the sacroiliac joint is often the result of a traumatic event, thus has a more sudden onset than facet joint or discogenic pain and is transmitted through the ventral rami of the L5-S2 for the anterior and the lateral branches of the dorsal rami of the S1-S4 nerve roots for the posterior part of the respective joint (16). A CT scan is usually preferred for sacroiliac pain with traumatic etiology. However, an MRI finds its usefulness when diagnosing inflammatory sacroiliac joint pain, such as in sacroiliitis resulting from spondyloarthritis. A study by Hangai et al. (17) has shown a correlation between the intensity of the signal on MRI and the symptoms of sacroiliac pain in patients experiencing the condition. In patients with non-inflammatory sacroiliac joint pain, readily available ultrasound evaluation of the long posterior sacroiliac ligament could reveal its thickening, soft tissue edema, and pathological transformations (18). The same authors showed in another clinical study that ultrasound changes in the attachment of the lumbar erector spinae muscles are associated with lumbosacral pain syndrome (19).

### Failed back surgery syndrome

This condition has a multifactorial genesis, being the result of both patient psychosocial factors, such as psychiatric comorbidities and bad habits like smoking and alcohol consumption, as well as intraoperative factors. Such are surgery at the wrong segment, an insufficient number of levels, or an inadequate technique for the respective case. Postoperative factors might be pointed out, such as recurrence of the condition and adjacent segment disease (20, 21). The condition presents either with exacerbation of the current symptoms or with the apparition of new ones. In the latter case, “Post-surgical spine syndrome” is a suitable term. Patients >65 years are generally more susceptible and failed back surgery syndrome is present in close to 15% of patients (22). The condition comes with many limitations for the patients, such as difficulty in activities like traveling and social life, as well as everyday life activities. The diagnosis involves a thorough medical history and imaging diagnostics appropriate for the respective pathology (21).

### Spinal instability

The diagnosis of spinal instability is impeded mainly by the ambiguous nature of the symptoms associated with the condition, which are not easily distinguished through common imaging diagnostics, such as CT scans, MRIs, and radiographs (23). Nevertheless, this pathology is one of the leading causes of LBP in younger patients and is caused by the instability of a vertebral segment reacting to applied loads. Microinstability, which describes the pure motion syndrome with no morphological changes and lack of defined structural abnormalities, has recently gained popularity. For a pathophysiological context, the lack of stabilization of the spine, usually applied by the segmental muscles generally inserted in it, provokes compensation by the trunk muscles. The range of motion of the spine is preserved. However, a painful arc is present, and erecting the body from a bent-over position is hindered. Single photon emission tomography could detect facet joint lesions. À diagnostic block in this situation could help differentiate the conditions (23).

### Acquired and congenital structural abnormalities

Acquired structural changes, such as spondylolisthesis and spondylolysis, have been debated regarding LBP. We have identified two systematic reviews that evaluated the association between the conditions mentioned above and LBP, showing no statistically significant correlation between the presence of the conditions and symptomatic LBP (24, 25). According to some authors, a relation has been identified between lumbar spondylolisthesis and lumbar spinal stenosis, an actual cause of LBP. Yet, no exacerbation of symptoms was demonstrated with varying levels of disc slippage (25).

On the other hand, scoliosis patients often experience pain at the curve’s apex and the inner side of the thigh – cruralgia. The lumbar and thoracolumbar curves are generally more painful than the thoracic curves, and the rotatory olisthesis – the lateral rotation of one over the other, has been identified as one of the major causes of said symptomatology (26). A study comparing the pain in left- and right-convex degenerative lumbar scoliosis in the context of the location of the pain areas found no significant difference between the two groups regarding location and pain severity. Nevertheless, a heat map was created with patients’ data, indicating that LBP was centrally located for most patients, regardless of whether they were left or right (27). Non-specific LBP differed in patients with scoliosis and was more inclined toward either side than in a non-scoliotic control group, in which the patients showed a more centralized pain pattern. Scoliosis patients also had differences in mobility and back muscle strength (28). Additionally, one paper was identified, which measured the changes in experienced pain throughout several different periods preceding the moment of the study and the accompanying degrees of insomnia and depression. Patients with current back pain reported daytime sleepiness and insomnia at higher levels, and those with chronic back pain had moderate depression in addition to insomnia and daytime sleepiness (28).

Lumbosacral transitional vertebrae, otherwise known as Bertolotti’s syndrome, is a highly prevalent anatomical variant in which a sacralization of the L5 or a lumbarization of the S1 is observed. In the former condition, the fifth lumbar vertebra adopts some characteristics of the sacral vertebrae. In the latter, the first sacral vertebra takes on the characteristics of the lumbar vertebrae (29). The literature is uncertain about the concrete connection between the condition and LBP. However, certain types of the condition were more strongly associated with LBP, such as type 2 – pseudoarticulation type with enlargement of the transverse process with pseudoarthrosis, and type 4, in which the transverse processes on one side were pseudoarticulated and on the other were fused (29).

The congenital fusion of vertebrae is most commonly localized in the cervical, followed by the thoracic and lumbar spine, diagnosed through a CT or an MRI scan. Nevertheless, a case report was identified of a patient with fusion vertebrae experiencing chronic low back pain. The authors hypothesized that narrowing the intervertebral foramen could be the source of the symptoms that arise with certain specific postures (30).

A hemivertebra is a condition where a vertebra is not fully formed, which causes a deformation of the physiological structure of the spine. Depending on the part of the anatomical structure that lacks development, the condition can present with kyphosis, lordosis, or scoliosis. The primary symptom is usually a noticeable trunk deformation, causing a cosmetic defect, pain, and neurological symptoms, such as gait and urinary disturbances. The condition can be diagnosed with a plain X-ray, yet more advanced techniques, such as CT and MRI scans, are helpful for therapeutic clarification (31).

## Non-mechanical or systemic conditions

**FIGURE 2:**
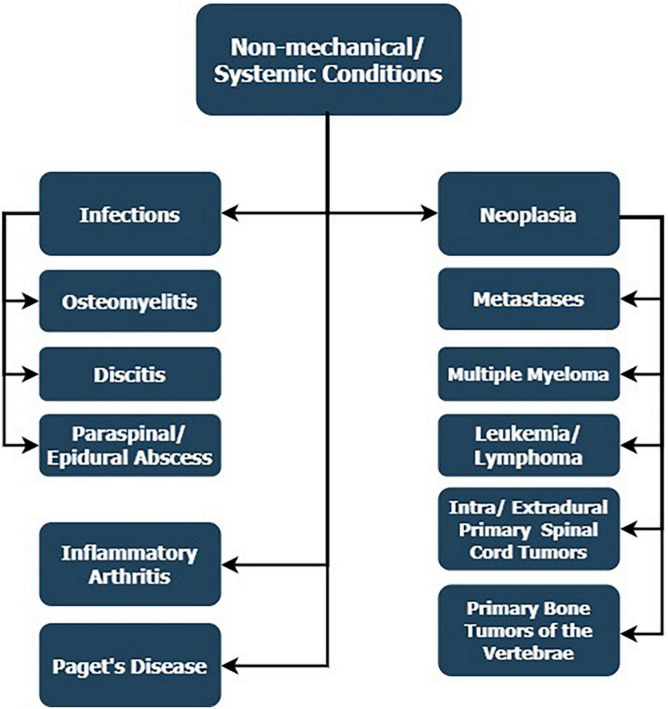
Non-mechanical reasons for LBP, whose development has not necessarily started in the spine’s components but is linked with the spine through dissemination or systemic conditions that engage it.

The non-mechanical causes of LBP find their genesis in systemic conditions, whose development has not necessarily started in the spine’s components (Figure 2). They are linked with this structure through dissemination or are systemic conditions that engage it. Additionally, the symptoms of engagement of the spine are usually accompanied by other manifestations of systemic diseases.

### Infections

The vertebral osteomyelitis is most commonly caused by a hematogenous disseminated Staphylococcus aureus or coagulase-negative staphylococci in exogenous osteomyelitis in spinal surgery. Tuberculosis is rare but should always be under suspicion. About 5% of osteomyelitis cases involve the posterior vertebral structures. More than 90% engage the vertebral body with the possibility of dissemination to adjacent structures, such as nerve roots, the epidural and intradural spaces, ligaments through the rich arterial web surrounding the vertebral bodies and toward the spinal column through retrograde dissemination through the Batson venous plexus (32). Spinal instrumentation surgery is a common predisposing factor for vertebral osteomyelitis (33). All immunodeficiency disorders also increase the risk of developing vertebral infections. The typical symptoms include back pain, initially non-focal, which later localizes over the affected area, and fever, although not in every patient. The pathology is most commonly located in the lumbar spine, followed by the thoracic and cervical segments. Thus, the condition is essential in the differential diagnosis of low-back pain, as it can be life-threatening (32).

As for the diagnosis, the imaging and the laboratory findings are crucial, with the MRI being the imagery of choice with more than 90% accuracy. The deviations to look for are decreased T1- and increased T2-weighted signals at the area of the infection. As for the complete blood count, the erythrocyte sedimentation rate and C-reactive protein have a 94% sensitivity (32). The diagnosis of spondylodiscitis – an infection of the intervertebral disc as well as the adjacent vertebrae can also be conducted through the use of radionuclide testing in specific cases, such as in evaluating the treatment response or generally cases when MRI is inapplicable. Fluorodeoxyglucose-18F has shown promising results, as it allows differentiating between infectious and degenerative endplate disease with high sensitivity (34). It might be helpful when assessing inflammatory, neoplastic, and traumatic/osteoporotic diseases.

Spondylodiscitis can cause a paraspinal abscess in the spinal region when the infection spreads to nearby tissues such as muscles and connective tissues of the vertebral column (35). Of particular note are psoas abscesses with genesis from hematogenous dissemination or adjacent to spondylodiscitis and infections in the abdominal cavity. They directly involve the paraspinal muscles and the lumbosacral plexuses. The abovementioned conditions have to be differentiated from epidural abscess, which is located in the spinal epidural space and is typically the result of a hematogenous dissemination of a bacterial agent from a remote location (36). One of the significant presenting symptoms of both infection types is LBP. The differentiation is based on the medical history – spondylodiscitis in the case of a paraspinal abscess and a systemic condition (e.g., bacteremia, immunosuppressed state) in the case of an epidural abscess, combined with an MRI scan for the more exact localization of the pathology (35, 36).

### Neoplasia

A lesion in the spinal column has the potential to produce LBP since it is usually the initial symptom in these cases, with 90% of spinal lesions being of a metastatic origin, most commonly from the breast, lung, and prostate (6). The complaint is usually at the level of the lesion. However, nerve involvement through compression could lead to a potential dermatomal distribution (37). Differentiating between causes through the correct imaging diagnostic, such as an MRI, is critical for treating the conditions since the patient could present with a more mainstream symptom of the spine, even when the primary tumor is yet to be found (38). Nevertheless, special attention should be placed on cases with known cancer that present with newly acquired back pain. A metastatic lesion is a common reason for a pathological fracture since the tumor cells are a source of osteoclastic and osteolytic activity, in which case the patient could present with excruciating axial and radicular pain (6, 37, 38).

The previous applies as well to multiple myeloma, which could progress asymptomatically until the occurrence of LBP due to a fracture. For the diagnosis of multiple myeloma, an MRI is not necessarily the optimal method, especially in cases where the bone marrow doesn’t present with tangible enough differences. In these cases, the CT and especially the SPECT scans are preferred since they more accurately capture the osteolytic process of the affected bones, combined with a blood lab analysis of the cell count and the elevated levels of globulins (39, 40). Nowadays, the differential diagnosis between a metastatic spine lesion and multiple myeloma is facilitated by machine learning algorithms that identify the features in an MRI to prioritize when examining (41).

As for differentiating between various types of malignancies affecting the spine, such as lymphoma and leukemia, for which a primary presentation in the axial skeleton is rare, the blood smear and complete blood count are of essential importance. The imaging diagnostic is a good addition for localizing the lesion and excluding other symptom causes. The presentation of leukemia primarily with LBP is more common in the pediatric population than in the adult population (42). The condition can cause pathological fractures with pain locally as well as compression of the nerve roots, which radiate toward the legs. The literature on the subject is scarce, consisting mainly of singular case reports (42, 43). One study showed 37 spinal lymphomas, where the lumbar region was the second most common localization with 10 cases, and pain was one of the significant presenting symptoms of the patients (44).

The intradural-extramedullary spine tumors are the second in frequency, following the extradural ones. The most common types are the schwannomas, followed by the meningiomas (45). The tumors can arise in each spine segment and are generally differentiated by their specific MRI findings (46, 47). Their initial symptom usually is axial or radicular pain, as well as sphincter and erectile dysfunction and paraparesis. The dumbbell appearance on imaging studies is more typical for schwannomas. On the other hand, a typical finding in meningiomas is the vivid enhancement when contrast is applied in combination with the characteristic dural tail of the tumor. The specific filum terminale ependymoma is also classified in this category. On T2 weighted MRI, the lesion is hyperintense and is typically well enhanced by contrast medium.

Intramedullary tumors, mainly comprised of ependymomas and astrocytomas, might be found in every spinal cord segment (46). Among the two, the ependymomas are more common in the spinal cord’s terminal parts, and the astrocytomas are more frequent at the thoracic level. Additionally, hemangioblastomas are the third entity on the list of intramedullary tumors, followed by metastases. On MRI, the astrocytomas tend to form syrinxes in the spinal cord and often span 5-6 segments, whereas the ependymomas present with a focal enlargement spanning around 3-4 segments and growing slowly and encapsulated. Angiography is a good examination for hemangioblastomas because it allows for assessing the feeding and draining vessels of the highly vascularized lesion. The most common symptom for these lesions is pain, which typically worsens during nighttime and could be radicular at the affected segment or distal with a neuropathic pattern. Furthermore, many neurological signs and symptoms can be present, caused by the tumor compressing and irritating motor or sensory nerves, such as gait disturbance, ataxia, paresthesias, as well as urinary disturbances.

### Chronic inflammatory conditions

Axial spondyloarthritis, or ankylosing spondylitis and Bechterew’s disease, causes inflammatory back pain, characterized by pain with an insidious onset before the age of 45 years, worse at night and during rest, with partial improvement during movement (48). The chronic inflammatory process causes ossification of the discs and ligaments and ultimately leads to fusion, which gives the spine the characteristic bamboo shape. To diagnose the condition early on, both an MRI and testing for HLA-B27 are performed. The prominent MRI feature is sacroiliitis, which may or may not be radiographically present, as well as the spine’s inflammation and the ligament attachment sites. The X-rays and CT scans are typical in the advanced stages of the disease. The HLA-B27 is positive in a large part of the population. However, its presence or absence does not rule out or confirm the diagnosis with complete certainty (48).

Even though rheumatoid arthritis is strongly associated with peripheral joint involvement, a recent study found that poor control of the disease was associated with worsened LBP in the long run (49). Nevertheless, more research is needed to explain the connection between the two conditions more thoroughly.

Forestier disease, or diffuse idiopathic skeletal hyperostosis, is characterized by the ossification of spinal ligaments and entheses (50). The excessive osseous structures created by the condition render the patient more prone to compression of nerve structures and secondary injuries in minor trauma. A study in 1989 found through a survey that among 106 patients with the condition, low back pain was not more common than the presence in the general population (51). Thus, the disease is still debatable as a cause of complaints.

In Paget’s disease, the spine is subject to abnormal bone growth caused by an upregulation of the osteoclastic activity, followed by excessive osteoblastic compensation, which leads to inadequacy in the size and shape of the affected bone. Regional pain is a typical presentation. The diagnosis is based on a CT scan of the bones or a radiograph, combined with alkaline phosphatase in the blood, hyperuricemia, and several other factors of the urine analysis (52).

## Referred pain in visceral diseases

**FIGURE 3:**
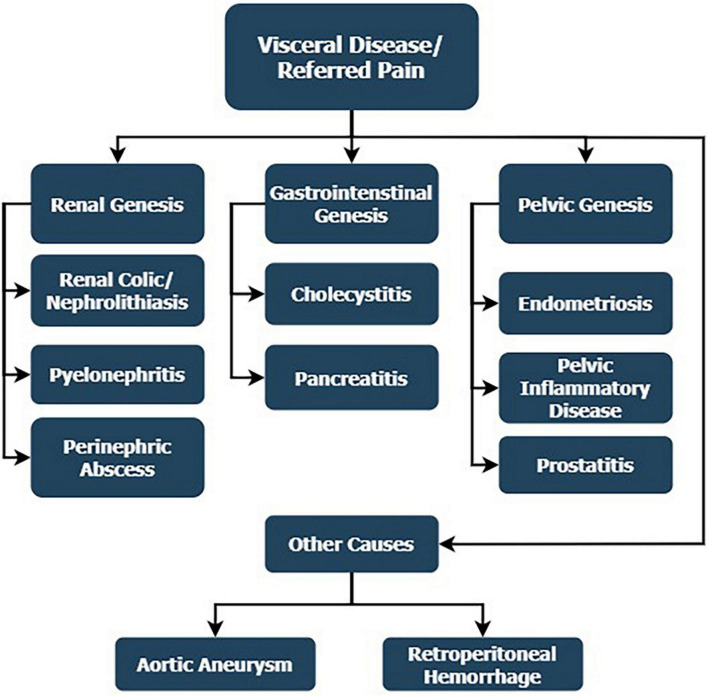
Referred low back pain in visceral diseases, which has little or nothing to do with the spine itself, and the primary cause of the symptomatology is usually a condition of a specific visceral organ or generally a process of the abdominal or pelvic cavities.

Referred pain is related to one that is felt in the lower back and has little or nothing to do with the spine itself (Figure 3). The primary cause of the symptomatology is usually a condition of a specific visceral organ or generally a process of the abdominal or pelvic cavities.

### Renal causes

Acute pyelonephritis is a frequent cause of flank pain, which can be mistaken for LBP of spinal genesis (53). Etiologically, the condition is caused by bacteria, most frequently gram-negative E. coli, which adhere to the renal parenchyma and cause an inflammatory response. The diagnosis is based on urinalysis plus ultrasound when available. However, the negativity of the latter does not exclude the presence of the disease in its acute form. The condition is further characterized by unilateral costovertebral angle tenderness, typically over the affected kidney. High fever is one of the differentiating symptoms between acute pyelonephritis and acute renal colic, which also causes the characteristic flank pain. Yet, this time, the symptom radiates toward the groin and is pulsatile since the peristalsis of the urethral muscles remains active (7, 53, 54). Overall, the pain during renal colic is reported as more excruciating. Pathophysiologically, it results from the stretching of the renal capsule caused by the retention of urine before the level of obstruction. The diagnosis is based primarily on a CT scan, which is the method of choice, and ultrasound, which visualizes hydronephrosis. Radiography is still helpful since some stones are radiolucent (54).

Nevertheless, flank pain could also be the leading symptom in a perinephric abscess, radiating both to the groin and the leg imitating radicular genesis. The condition could result from a local infection or a hematogenous spread, affecting the renal capsule and Gerota’s fascia. The standard for diagnosing the pathology is the contrast-enhanced CT scan, which gives additional information about the spread of the condition to adjacent structures (55).

### Gastrointestinal causes

As for the gastrointestinal causes, acute cholecystitis and pancreatitis could produce pain. However, it radiates more toward the mid back and upper abdomen than the lower back (56, 57). The history of these patients usually includes the consumption of greasy foods in cholecystitis, which provokes gallbladder emptying and subsequent colic, whether in the presence of gallbladder stones or chronic alcohol consumption in the pancreatitis group. Nevertheless, gallstones could produce the discussed complications through obstruction of the common ducts. Both conditions are visualized through plain CT scans and ultrasound in the case of cholecystitis. The diagnosis is supported through a liver enzyme check-up (56, 57).

### Pelvic disorders

Pain originating from the pelvic region is anatomically close to the lower spine. Endometriosis is one of the most common reasons for non-spinal LBP (7). The condition causes a painful inflammatory reaction, which could exacerbate the symptoms through the spread toward adjacent structures. It produces pain in the pelvic region that could be mistaken for LBP, as irradiation toward either of the legs does occur (58). Either ultrasound or an MRI is employed. The symptoms of dyspareunia and irregularities in the menstrual cycle support the diagnosis (59). Pelvic inflammatory disease in women is an ascending infection most typically caused by Neisseria gonorrhoeae or Chlamydia trachomatis, which, aside from pelvic and abdominal pain, presents with vaginal bleeding and dyspareunia (60). The bacteria can be identified through pelvic culture, and imaging diagnostics such as an MRI are reserved for evaluating any potential complications of the condition. As for the male population, prostatitis could be a cause of pelvic pain accompanied by some urinary as well as general symptoms of infection. Urinalysis is assessed for evaluation of the involved pathogen (61).

### Other causes

Both abdominal and LBP are some of the most common symptoms of retroperitoneal hemorrhage, which could happen both as a result of a traumatic event or iatrogenic, following surgery or anticoagulation therapy (62). The symptoms are generally vaguer when compared to the insidious nature of the condition with high mortality. The presence of a hypovolemic shock, which renders the patient hemodynamically unstable, combined with a thorough history of the patient and a CT scan, should be used for the differential diagnosis of the condition with other types of LBP. CT scans are beneficial for patients who have not undergone trauma since such an event usually facilitates the diagnosis (63).

Additionally, an abdominal aortic aneurysm could potentially cause LBP through compression of nearby structures as well as during a rupture (64). The aorta is adjacent to the spine, so enlargement of the wall could affect the surrounding structures and provoke complaints. Nevertheless, the typical symptoms associated with the condition are not always present. Therefore, the imaging diagnostic, mainly through ultrasound, is prioritized (65). Smoking and hypertension are the predisposing factors for the formation of the aneurysm. However, after that, the symptom could shift to hypotension, and both are potentially a part of the patient’s presentation (65).

## Other

Fibromyalgia is another condition that is associated with chronic LBP and cannot be classified in the previous categories. The diagnosis is often controversial and mainly made in rural areas, more commonly in women. The back pain associated with fibromyalgia does not differ noticeably from alternative types of chronic widespread pain. The etiology of the condition has been strongly linked with psychosocial factors. Usually the patient complains of additional disturbances, such as poor concentration, sleep, and memory, as well as irritability (66).

## Conclusions

The diverse etiology of conditions contributing to the symptoms of LBP renders the diagnosis difficult to determine. The fact that not all of the conditions are attributable to the spinal structures themselves, and certain ones having been scarcely reported in the literature, further aggravates the confusion around the subject. In addition, many cases of LBP are that of non-specific pain, which cannot always be attributed to any apparent deviation from normal physiology. Having the correct imaging diagnostic conducted for the patient can positively impact the differentiation between the diverse types of pathology. Still, a certain number of conditions present with seemingly ambiguous findings. Thus, a sufficient combination of the proper blood tests, imaging studies, medical history, appropriate and meticulous clinical examination focused on the patient problem is imperative for the case. A good knowledge of the underlying causes and the differential diagnoses could facilitate this process.

## Authors contributions

DF: Conceptualization, Methodology, Supervision, Writing – review & editing. DY: Resources, Writing – review & editing, Supervision. MT: Formal Analysis, Methodology, Visualization, Writing – original draft.
